# Quality of surgical management of placenta accreta spectrum in a tertiary center in Sri Lanka: baseline study for quality improvement project: problems and solutions

**DOI:** 10.1186/s12884-022-04840-7

**Published:** 2022-06-23

**Authors:** Vindya Wijesinghe, Mohamed Rishard, Sriskanthan Srisanjeevan

**Affiliations:** 1 Resident Obstetrician and Gynaecologist, Ministry of Health, Nutrition and Indigenous Medicine, Colombo, Sri Lanka; 2 Department of Obstetrics and Gynaecology, Faculty of Medicine, Consultant Obstetrician and Gynaecologist, University of Colombo Sri Lanka, Colombo, Sri Lanka; 3 Registrar in Obstetrics and Gynaecology, Post Graduate Institute of Medicine, Colombo, Sri Lanka

**Keywords:** Placenta accreta spectrum, Physical morbidity, Psychological morbidity, Impact of life event scale- Revised (IES-R) questionnaire, Short form health survey-36(SF-36) questionnaires

## Abstract

**Introduction:**

Placenta accreta spectrum (PAS) is associated with a multitude of maternal and fetal complications. Events related to its management have resulted in significant psychological morbidity, with lifelong consequences which warrant continuous support to cope with their lives. The objective of the study is to highlight the importance of multidisciplinary holistic care and explore room for improvement in the provision of care for women with PAS.

**Methods:**

Our study was conducted on deliveries complicated with PAS from January 2019 to June 2021. 27 women were diagnosed with PAS during this period and received treatment. Impact of life event scale- revised (IES-R) and short form health survey-36(SF-36) questionnaires were administered to assess the impact of PAS on their lives. In depth interviews were conducted with the women. A multidisciplinary team meeting was later conducted to formulate a comprehensive care plan for women with PAS.

**Results:**

The response rate was 96.2%. Mean age of the sample is 34.1 years (SD 4.3). Interval to current pregnancy varies from 0.6 years to 10 years with mean of 4.6 years. Mean gestational age of diagnosis of PAS was 25.4(SD 6.7) weeks. The care bundle provided for women with PAS was evaluated in all cases.

Surgery was conducted electively in 82% of patients. Blood transfusions were noted in 85%. Mean pre-operative stay was 9.5 days (SD 8.3) and mean post-operative stay was 8.8 days (SD 8). Total hospital stay ranged from 6 to 48 days (mean 19.5 days, SD 11.4).

IES-R scores were significant in 4/26 patients. There was no correlation with the interval from the surgery with any of the subscales or with the total scores. The lapsed time after the surgery had a significant correlation with physical functioning and pain domains of the SF-36 questionnaire.

Description of the experience, loss of femininity with loss of the uterus, concerns and fears about the future and measures to improve the quality of care were the themes identified and described.

A multidisciplinary team meeting, consisting of consultant obstetricians performing surgery for PAS, anesthetists, hematologist, transfusion medicine specialist, urologist, physiotherapist, nutritionist and nursing officers from ICU and wards, was held and their contributions helped to map out a definitive care plan.

**Conclusions:**

PAS is associated with long term physical and psychological morbidity. Continuous support through quarterly clinic visits and telephone conversations may alleviate the psychological trauma. However, some physical disabilities may be lifelong and life changing. Importance of reducing primary caesarean section and promoting trial of labour after caesarean delivery should be promoted among patients and caregivers.

**Supplementary Information:**

The online version contains supplementary material available at 10.1186/s12884-022-04840-7.

## Introduction

Placenta Accreta Spectrum (PAS) is a complication that commonly arises following a previous caesarean section (CS) or uterine surgery [1–3]. The placenta is inseparably attached to the myometrium with varying depths of invasion. Increasing depths of invasion is associated with increased rates of complications which can be life-threatening and life-altering. PAS is associated with antenatal, intrapartum, and postpartum complications that have increased maternal and foetal morbidity and mortality [1–3].

Delivery of the fetus is always by a caesarean section (CS) by 35 to 36 weeks of gestation as further prolongation of pregnancy could lead to catastrophic obstetric haemorrhage. An obstetric hysterectomy is often performed in the presence of massive obstetric haemorrhage [4, 5]. But certain percentage of women with PAS undergo emergency hysterectomy [6, 7]. PAS is closely associated with placenta previa, and this may give rise to one or more episodes of antepartum haemorrhage that require prolonged hospital stay [6, 7]. A series of stressful events may occur from the point of diagnosis till discharge, which may traumatize the patient and their family members [8, 9].

Reducing the rate of CS is a global requirement [10, 11]. However, CS rate has risen in low- and middle-income countries( LIMC) during the past two decades including in Sri Lanka [12]. PAS is a recognized complication of caesarean sections and the most dangerous. It is apparent that countries with high CS rates should take initiatives to reduce primary CS rates, but also be equipped to handle the burden of rising number of women with PAS.

Women with PAS tend to stay in the hospital for longer periods for preoperative optimization, undergo multiple counselling sessions prior to surgery, and attend multidisciplinary team (MDT) meetings [8]. Surgery is performed under general anesthesia (GA) where she loses the opportunity to be consciously with the newborn. Many patients undergo hysterectomy, with loss of fertility and perceived loss of femininity. Some women require repeat or interval surgical interventions to treat the complications that may arise from primary surgery.

Surgical techniques and surgical outcomes have been studied and prediction models have been developed in the recent past [13–17].Even though the psychological burden of the diagnosis of PAS is apparent, psychological morbidity that these women undergo is often underestimated and not addressed [18, 19].

The diagnosis of PAS and its aftermath are traumatic. The psychological ramifications are neither documented nor addressed. Anecdotal evidence suggests that woman suffer from long term psychological and physical issues. Thus, PAS can give rise to post-traumatic stress disorder (PTSD), and this often goes undiagnosed and untreated [20].

PAS can be devastating to the woman and the family. In this study we hope to describe the impact PAS has had on the lives of women in a quantifiable manner.to suggest a holistic approach.

## Method

The study was conducted at De Soysa Hospital for women (DSHW), Colombo, Sri Lanka. which is the second oldest maternity hospital in Asia. It is a tertiary center of care for women with high-risk pregnancies, including those complicated with PAS.

Our study was carried out on deliveries conducted from January 2019 to June 2021.This was a mixed method study. Phase 1 comprised of an audit to examine whether the standards were followed in the surgical management of women with PAS; administration of two validated questionnaires to assess the quality of life following the surgical management of PAS via an in-depth interview. Phase 2 involved summarizing the findings of phase 1 at a multidisciplinary meeting to formulate a comprehensive care plan to improve outcomes.

Patients’ contact details were obtained from the admission registry of the obstetrics ward. Clinical records were traced, and pre-operative and intra-operative details were gathered. Patients were contacted over the phone and via email where possible. Information sheet and consent form were elaborated and appointments were given after consent.

All women who had PAS were included in the study.

### Clinical Audit

An audit was conducted to assess whether the standards of PAS care bundle had been followed in the management these women. Clinical records of these women were traced, and data was entered in an audit pro forma.

### Operative outcomes

Clinical records were used to gather information regarding the outcomes of the procedures and recorded in data collection sheets.

### Validated questionnaires

#### Impact of life Event scale -Revised (IES-R)

Sinhala and Tamil versions are validated. However, the Sinhala version is not published yet and the questionnaire was obtained after a personal communication with the author.

The scale contains 22 questions and patient responses are obtained on a scale from 0-4. The option patient select will be noted down on the scale. IES-R is a commonly used questionnaire for assessment of impact following a traumatic event. The twenty-two items are rated on a 5-point scale ranging from 0 ("not at all") to 4 ("extremely"). The IES-R yields a total score (ranging from 0 to 88) and subscales intrusion{(INT) (unavoidable memories, flashes, and strong thoughts about the particular event)}, avoidance{(AVD) (avoiding the objects, places, people which reminds you about the event)} and arousal{(HYP) (uncontrollable awake state which prevents from resting and reminds you about the particular state)}. The “Intrusion’ subscale is assessed by the items 1,2,3,6,9,14,16 and 20. The “Avoidance” subscale is assessed by the items 5, 7, 8, 11, 12, 13, 17 and 22. The “Arousal” subscale is assessed by the items 4, 10, 15, 18, 19 and 21. Scores for the IES-R are meaningful with a cumulative score of 24 or more suggesting there is significant impact on the quality of life from the event. PTSD is a clinical possibility from a life threatening and life altering event. High scores would imply a diagnosis of partial PTSD with a score of 33 or more diagnostic of PTSD.

#### Short-form health survey – 36 (SF- 36)

The validated Sinhala and Tamil versions of the questionnaire was used in this study.

SF-36 scoring measures eight health concepts: physical functioning, bodily pain, role limitations due to physical health problems, role limitations due to personal or emotional problems, emotional well-being, social functioning, energy/fatigue, and general health perceptions. It also includes a single item that provides information on the perceptible change in health compared to a year ago.

Scoring of the 36-item Health Survey is a two-step process. Numeric values were assigned to each answer. Each item is scored on a range of 0 to 100. The scores for each health domain were added and an average score was obtained. A higher score represents a more favorable state of health.

### In- depth interview 

An in-depth interview was conducted using an interviewer guided questionnaire, under the following key themes.

- 1. Description about the experience.
- 2. Loss of the uterus.
- 3. Concerns and fears about the future.
- 4. Measures/suggestions to improve quality of the service provided.

### Multidisciplinary team meeting

The findings of the study were presented at a multidisciplinary team meeting consisting of five consultant obstetricians who are performing surgery for PAS, two consultant anesthetists, consultant hematologist, transfusion medicine specialist, urologist, physiotherapist, and nutritionist).Ideas and suggestions to improve the service for women with PAS were discussed. An ideal management protocol and a comprehensive care plan were finalized at the end of the meeting and a decision to prepare a checklist for provision of care for women with PAS.

Paper records were locked in a cabinet in a secured room. Electronic data were kept under password protection. All data will be kept for three years after the official end date of the study. Confidentiality was maintained and patients’ private details were not divulged when disseminating the results of the study. All concerns of the women involved were addressed and those who had medical issues were referred to the necessary disciplines. Furthermore, a helpline was established to address future health issues of the women in the study.

Data were analyzed using SPSS software(Version 23). Mean value was used for describing the central tendency of observation for each variable. *p* value of less than 0.05 was regarded as statistically significant.

## Results

Twenty-seven women had undergone surgery for PAS at the study center within the last two and a half year. 26 of those women responded to the invitation to take part in the study, with a response rate of 96.2%.

Mean age of the sample is 34.1 years (± 4.3) with a minimum age of 23 years, to a maximum age of 45 years. Mean interval from surgery to data collection was 13.8 months (± 7.9). 7 patients were recruited within the first six months of the surgery.

Most women were in their second pregnancy. There was one primigravida with a previous second trimester miscarriage complicated by chorioamnionitis, which resulted in an evacuation of retained products of conception (ERPC). Six women with one previous caesarean delivery, and four women with three previous caesarean deliveries were also included in the study.

Out of the 26 women who had caesarean sections (CS), 15 of those were performed as elective procedures.

Four CS sections were performed for presumed fetal macrosomia but only one baby was over 3.5 kgs. Whilst all other babies were less than 3 kgs. Three ECS were performed for oligohydramnios. Another two sections were done due to high unengaged head. None of the mothers who underwent an elective caesarean section were offered trial with labour whilst a maternal request for caesarean section was accommodated in two women.

Eleven caesarean sections were performed as emergencies (EmCS), 45.4% (5/11) for perceived fetal distress, 27.2% (3/11) for lack of progression, 9% (1/11) for breech in labour, 9% (1/11) for reduced fetal movements and 9% (1/11) for antepartum hemorrhage (APH).

Nineteen patients had repeat elective caesarean section (RECS). 84.2% (16/19) underwent RECS due to past Sect. 10.5% (2/19) was given a trial of labour after caesarean (TOLAC) but developed lack of progress. 5.2% (1/19) had emergency repeat CS due to past section in labour and she was not offered TOLAC.

Interval to current pregnancy varied from 0.6 years to 10 years with mean of 4.6 years. Mean gestational age of diagnosis of PAS was 25.4(SD 6.7) weeks. Minimum period of amenorrhea (POA) at diagnosis was 6 weeks and maximum POA of diagnosis was 35 weeks.

40.7% (11/27) of women were referrals to DSHW from local hospitals from across the country59.3% (16/27) of women were initially registered at DSHW. 37% (10/27) patients experienced one or more episodes of 1^st^ trimester bleeding and one patient had recurrent bleeding for 2 months. 18.5% (5/27) had a history of APH. Only one patient had a massive APH that necessitated blood transfusions prior to surgery. Hospital admissions before the admission for surgery were seen in 20 patients with mean admission rate of 1.6 times. Whilst one patient had sought admission to the hospital on six separate occasions.

All deliveries were attended by the consultant obstetrician and consultant anesthetist. Blood and blood products were ordered for all women. All women were booked for immediate post-operative care in the surgical intensive care unit (level 2 critical care bed). Surgical referral was done prior to surgery in 24/27 cases. Additional input concerning patient care was obtained from the Haematologist (96.3%), Transfusion medicine specialist (88.9%) and physiotherapist (77.8%) in most cases.

The advice of the clinical nutritionist was obtained in the management of 15 women during their post-operative period.

The Radiologist was involved in PAS mapping using ultrasonography in all the cases, but none of them had been offered an MRI.

Ten out of 27 patients had some antenatal complication. One patient had history of depression and antenatally she was referred to the psychiatrist.

Type and methods were used during the surgery is indicated in Table 1.

**Table 1:** Methods used in the surgery and ligation of resection of tubes during conservative surgery

		**Number out of 27**	**Percentage %**
**Type of the surgery**^**a**^	Emergency surgery	5	18%
	Elective surgery	22	72%
**Type of skin Incision**	Midline	21	77.70%
	Pfannensteil	6	22.20%
**Type of uterine incision**	Classical	19	70.30%
	Transverse	8	29.70%
**Type of surgery**^**b**^	Total hysterectomy	17	62.90%
	Subtotal hysterectomy	1	3.00%
	Conservative	9	33.30%
**Contraception for conservative surgery**^**c**^		5 out of 9	55.50%

^a^Type of surgery according to the urgency eg: elective or emergency

^b^Type of surgery according to the procedure

^c^Number of patient who received ligation and resection of tubes after the conservative surgery

Average POA at the time of emergency surgery 33 weeks and 4 days to 36 weeks and 5 days. Average POA at the time of elective surgerywas 34 weeks and 2 days to 38 weeks and 5 days.

Emergency surgeries were conducted for onset of labour (4/5) and APH (1/5).

Other post-operative complications are indicated in Table 2.

**Table 2:** Surgical complications with number of patients and percentage

Post operative complications	Number of patients	%
**Blood transfusions**	23	85.1
**Intensive care unit (ICU) stay**	27	100
**Mild Disseminated intravascular coagulation (DIC)**	1	3
**Bladder injury**	8	29.6
**Abdominal drain more than 48 h**	2	7.4
**Bladder haematoma**	3	11.1
**Prolonged irrigation of the bladder more than 48 h**	7	25.9
**Bladder fistula**	1	3.7
**Paralytic ileus**	2	7.4
**Post-partum psychosis**	2	7.4
**Anaphylaxis to blood products**	1	3.7
**Surgical site infection (SSI)**	4	14.8
**Repeat surgery for hernia**	1	3.7

All post-operative complications are presented in percentages

Mean estimated blood loss (EBL) was 1950 ml (± 1400), with a minimum blood loss of 200 ml and maximum of 6000 ml. 84.6% had been transfused with red cell concentrates (RCC) with mean of 3.8 L (± 2.5). Cryoprecipitate was given to 16 patients (42%). Mean ICU stay was 4 days (Range 1–10 days). One patient was kept on an abdominal drain for 2 weeks due to continuous high output. She was subsequently found to have a vesicovaginal fistula for which she was managed conservatively by the Urology team with an indwelling urinary catheter for 4 months

Three women developed surgical site infection (SSI) which resulted in a prolonged time for healing of 1–2 months. None of the women required readmission though frequent hospital visits were noted. Some patients had visited every other day for checkups and wound dressings. Two women exhibited symptoms of postpartum psychosis, one of which had had an antenatal history of depression. Both women received treatment and routine follow-up from the Psychiatrist.

Mean pre-operative duration of stay was 9.5 days (± 8.3) and mean post-operative duration of stay was 8.8 day (± 8). Total hospital stays ranged from 6 to 48 days (mean 19.5, ± 11.4). Intravenous antibiotics were administered for an average of 5.4 days (± 3). Oral antibiotics were given for an average of 4.9 days afterwards.

Average birthweight was 1.7 kg (± 338 g). 40.7% babies needed admission to the baby care units and 40.7% babies received phototherapy. Four mothers experienced issues with establishment of breast feeding and one baby had developed neonatal sepsis.

Four patients had clinically significant scores (Table 3). They were counselled and referred to the psychiatry clinic. Subscale scores were correlated with each other and the total scores. (Pearson correlation, two tailed test and significant level of correlation at 0.01 level). There was no correlation between the interval from the surgery with any of the subscales or the total scores.

**Table 3:** Responses to IES-R

Interval from surgery(months)	INT	AVD	HYP	TOTAL
2	6	8	0	14
26	2	1	0	3
21	0	0	0	0
3	15	15	12	42
6	1	0	0	1
14	0	0	1	1
10	3	5	3	9
10	9	15	10	34
20	1	0	0	1
24	6	7	2	15
26	5	8	3	16
12	4	5	1	10
11	0	2	0	2
4	4	7	4	15
5	0	1	0	1
14	4	0	3	7
4	0	0	0	0
11	5	10	0	15
22	5	3	2	10
10	11	5	8	24
19	10	18	4	32
3	3	4	0	7
22	14	17	7	38
19	5	4	1	10
19	10	9	3	22
24	6	6	2	14

*INT* intrusive sub scale, *AVD* arousal sub scale, *HYP* hyperactive. *Total* Summation of all subscales. Actual scoring given by the patients are presented under each subscale, Total score of the IES-R is presented under the "total score”

Table 4 illustrates means scores for each domain in SF-36 questionnaire of 26 patients.

**Table 4:** Heath related quality of life (SF-36)

SF-36 Domains	Mean	Std. Deviation
physical Functioning	79.6154	15.80652
Role limitations due to physical health	65.3846	41.87895
Role limitations due to emotional health	80.7615	35.49976
Energy/Fatigue	81.5385	17.2493
Emotional well-being	84	16.70449
Social functioning	78.6538	25.5659
Pain	66.7308	21.47987
General Health	71.2019	24.65887
Heath change	49.0385	24.98076

Mean scores of the eight domains in SF-36 for all respondents is presented here. In addition, health change compared to last year is presented in the last ro**w**

Pearson correlation with a level of significance at 5% (0.05) was calculated with the time lapsed from the surgery and each domain. The interval from the surgery had significant correlation with physical functioning and pain (0.05 and 0.003 respectively). However, the interval from the surgery had no correlation with any other domain. Every domain was correlated with each other, except the pain domain which was not correlated with role-emotional (0.082). Social functioning was not correlated with role-emotional domain (0.453) (Table 5).

**Table 5:** Correlation of SF-36 and IRES-R domains

		Physical functioning	Role limitations due to Physical problems	Role limitations due to emotional problems	Energy	Emotional wellbeing	Social function	Pain	General Health	Health Change	Intrusive Subscale	Avoidance Subscale	Arousal Subscale	Total score IES-R
**physical functioning**	Pearson Correlation	1	.674	.450^a^	.483^a^	.485^a^	.668	.707	.585	0.316	-0.35	-.425^a^	-.483^a^	-.443^a^
	Sig. (2-tailed)		0	0.021	0.012	0.012	0	0	0.002	0.116	0.08	0.03	0.013	0.023
	N	26	26	26	26	26	26	26	26	26	26	26	26	26
**Role limitation due** **to Physical problems**	Pearson Correlation	.674	1	.745	.665	.669	.482^a^	.553	.692	-0.033	-.524	-.516	-.540	-.552
	Sig. (2-tailed)	0		0	0	0	0.013	0.003	0	0.873	0.006	0.007	0.004	0.003
	N	26	26	26	26	26	26	26	26	26	26	26	26	26
**Role limitations due** **to emotional problems**	Pearson Correlation	.450^a^	.745	1	.442^a^	.459^a^	0.154	0.347	.549	-0.134	-0.267	-0.377	-0.238	-0.314
	Sig. (2-tailed)	0.021	0		0.024	0.018	0.453	0.082	0.004	0.513	0.187	0.057	0.241	0.119
	N	26	26	26	26	26	26	26	26	26	26	26	26	26
**Energy**	Pearson Correlation	.483^a^	.665	.442^a^	1	.963	.456^a^	.651	.677	0.12	-.665	-.643	-.686	-.703
	Sig. (2-tailed)	0.012	0	0.024		0	0.019	0	0	0.561	0	0	0	0
	N	26	26	26	26	26	26	26	26	26	26	26	26	26
**Emotional wellbeing**	Pearson Correlation	.485^a^	.669	.459^a^	.963	1	.461^a^	.645	.658	0.058	-.723	-.692	-.720	-.756
	Sig. (2-tailed)	0.012	0	0.018	0		0.018	0	0	0.78	0	0	0	0
	N	26	26	26	26	26	26	26	26	26	26	26	26	26
**Social function**	Pearson Correlation	.668	.482^a^	0.154	.456^a^	.461^a^	1	.568	.523	.444^a^	-0.373	-0.336	-.573	-.437^a^
	Sig. (2-tailed)	0	0.013	0.453	0.019	0.018		0.002	0.006	0.023	0.06	0.093	0.002	0.026
	N	26	26	26	26	26	26	26	26	26	26	26	26	26
**Pain**	Pearson Correlation	.707	.553	0.347	.651	.645	.568	1	.560	.423^a^	-.500	-.512	-.471^a^	-.530
	Sig. (2-tailed)	0	0.003	0.082	0	0	0.002		0.003	0.032	0.009	0.008	0.015	0.005
	N	26	26	26	26	26	26	26	26	26	26	26	26	26
**General health**	Pearson Correlation	.585	.692	.549	.677	.658	.523	.560	1	0.268	-.556	-.642	-.681	-.664
	Sig. (2-tailed)	0.002	0	0.004	0	0	0.006	0.003		0.186	0.003	0	0	0
	N	26	26	26	26	26	26	26	26	26	26	26	26	26
**Health change**	Pearson Correlation	0.316	-0.033	-0.134	0.12	0.058	.444^a^	.423^a^	0.268	1	-0.14	-0.11	-0.212	-0.163
	Sig. (2-tailed)	0.116	0.873	0.513	0.561	0.78	0.023	0.032	0.186		0.495	0.591	0.299	0.426
	N	26	26	26	26	26	26	26	26	26	26	26	26	26
**Intrusive subscale**	Pearson Correlation	-0.35	-.524	-0.267	-.665	-.723	-0.373	-.500	-.556	-0.14	1	.853	.826	.963
	Sig. (2-tailed)	0.08	0.006	0.187	0	0	0.06	0.009	0.003	0.495		0	0	0
	N	26	26	26	26	26	26	26	26	26	26	26	26	26
**Avoidance subscale**	Pearson Correlation	-.425^a^	-.516	-0.377	-.643	-.692	-0.336	-.512	-.642	-0.11	.853	1	.692	.939
	Sig. (2-tailed)	0.03	0.007	0.057	0	0	0.093	0.008	0	0.591	0		0	0
	N	26	26	26	26	26	26	26	26	26	26	26	26	26
**Arousal subscale**	Pearson Correlation	-.483^a^	-.540	-0.238	-.686	-.720	-.573	-.471^a^	-.681	-0.212	.826	.692	1	.872
	Sig. (2-tailed)	0.013	0.004	0.241	0	0	0.002	0.015	0	0.299	0	0		0
	N	26	26	26	26	26	26	26	26	26	26	26	26	26
**Total score of IES-R**	Pearson Correlation	-.443^a^	-.552	-0.314	-.703	-.756	-.437^a^	-.530	-.664	-0.163	.963	.939	.872	1
	Sig. (2-tailed)	0.023	0.003	0.119	0	0	0.026	0.005	0	0.426	0	0	0	
	N	26	26	26	26	26	26	26	26	26	26	26	26	26

^a^All significant levels are calculated using Pearson correlation at significance level of 0.05%

Eight out of 26 women reported that the surgery had affected their sexual life, with complains of less libido and less satisfaction. Six women also complained of dyspareunia. One woman had yet to recommence sexual activity even after 2 months since the surgery as she had lower abdominal pain.

7.7% (2/26)patients were not happy about the cosmetic outcome of the surgery. Dysmenorrhea, chronic pelvic pain (CPP) and back pain were experienced by 30.8% (8/26)following the surgery.

The economic impact of a life altering surgery cannot be understated. Two of the women were employed at the time of diagnosis and both resigned from their jobs promptly afterwards. The partners of five of the women incurred financial problems, either a loss or reduction of income, due to leave. The families of three women opted to rent a place closer to DSHW after being transferred from their local hospitals, incurring a heavier financial burden.

Two women out of who had uterine preserving surgery are expecting children. Out of 2 one is with past section and index midline laparotomy with classical incision expecting to undergo trial of scar as well.

### In- depth interview with patients

1. Description of the experience

Patients often used words such as “fear”, “frustration”, “near death” and “unforgettable” when describing their experiences. To most women, this was their most fearful experience in their life. Many were satisfied about the care they receivedand were grateful to the staff during the interview as well.

During the administration of IES-R, some described that they experienced all or most of the items following discharge, and the symptoms subsided with time. Some of the women unfortunately responded, “I try to avoid the hospital as it is too painful to me”.

2. Loss of uterus

Many were facing the emotional dilemma of the loss of their femininity due to the loss of the womb. Some were uncomfortable about “taking away the choice of having children”. Some expressed “part of my body is removed” exhibiting a sense of incompleteness.

One woman expressed that “Only my mother and I know, how I suffer from the feeling related to the event, I keep it as a secret from my husband. I feel that I am incomplete”.

Some of the women noted that if they had been well informed about the possible life-threatening complications of repeated caesarean delivery, they would have opted for VBAC.

3. Concerns and fears about the future

Many women expressed their concerns about hysterectomy. Many asked “does it affect my heath in long run” “Even though I am feeling okay now can I get problems due to removal of the womb, in the future ?”.

A small number of women had no clear idea of what had been removed, and they were eager to know about the preservation of their ovaries, the necessity of pap smears, whether the Fallopian tubes were removed or not, plausible causes for abdominal pain (that they were currently experiencing) and the possibility of ectopic pregnancies in the future. Some women inquired about the necessity of hormone replacement therapy after surgery.

4. Measures to improve quality of the services

Prolonged hospital stay was a concern for many women, and they suggested that “finding a way to reduce hospital stay” would benefit the whole family financially and psychologically.”

Delayed POA at time of diagnosis was questioned by some and they suggested that early diagnosis might have allowed them to prepare for the surgery.

All the women were happy about the pre-operative counselling they received with their family members. However, they had lot of unresolved issues before discharge. Pain management after discharge was not adequately addressed, and the women had to go to the local doctor to obtain medication for pain relief.

All the women appreciated the fact that follow-up after the discharge was as an outpatient, not requiring admission.

### Multidisciplinary team meeting summery

A zoom meeting was conducted with all consultant obstetricians who are performing surgery for PAS, consultant anesthetists, consultant hematologist, transfusion medicine specialist, urologist, physiotherapist, clinical nutritionist and nursing officers from ICU and ward. The following suggestions were submitted.

1. Consultant Obstetrician – It was decided to screen all the women with a previous caesarean delivery at 12 weeks and 20 weeks for PAS. Further, women presenting with 1^st^ trimester bleeding at earlier POA with a history of previous uterine surgery, are to be referred to a consultant obstetrician or consultant radiologist for further care.
  - Placental mapping should be done and drawn on the BHT. A repeat ultrasonographic examination must be performed before starting the surgery.
  - Surgery must be performed by two experienced consultant obstetricians (in collaboration with gynae-oncologist at DSHW). The training of junior consultants was emphasized and a decision to record all surgical procedures for PAS, with the consent of women, was taken.
  - An intraoperative ultrasound examination may be useful to delineate the placental edge and determine the best site for the uterine incision, which should avoid transecting the placenta.
  - Bladder dissection should be performed prior to uterine incision. A vertical uterine incision( classical) is performed, at least two fingerbreadths above the placental edge. Leaving a margin of myometrium between the placenta and uterine incision helps to prevent disruption of the placenta during opening or closing of the uterus.
  - After delivery of the infant, the cord is cut, the uterine incision is rapidly closed to decrease blood loss, and hysterectomy is performed.
  - Oxytocics should be initially avoided as it increases the chances of bleeding due to placental separation, however, it can be considered in cases where the placenta has either naturally, or accidently separated and conservative surgery is performed after resection of the attached segment.

Training session on ultrasonographic features of PAS will be conducted to all trainees and senior house officers in due course.

MDT meetings will be conducted for all women with suspected PAS to reduce pre-operative hospital stay and improve quality of care.. The meetings should involve women and their family and should adopt an individualised approach.

2. Consultant Anesthetist -. General anaesthesia is preferred with epidural analgesia for postoperative pain management. However, all patients will be required to undergo screening for other medical disorders. Full blood count, serum electrolyte, liver function test, renal function test, clotting studies and electrocardiogram should be arranged as outpatient to minimise hospital stay. However, further investigations should be considered in cases with diagnosed medical disorders (e.g. -heart disease). Such patients should be admitted to the hospital for evaluation.

Patients undergoing elective surgery for PAS should be received at the ICU prior to surgery for pre-operative preparation such as insertion of central venous line, arterial line, and epidural catheter.

- 3. Consultant radiologist- Though MRI facilities are currently not available at DSHW, it would be beneficial in mapping the extent of placental invasion in cases of bladder or bowel invasion, or if conversative surgery is planned. However, Ultrasonography has proven to be successful in the evaluation thus far. Interventional radiological methods are associated with increased rates of complications and currently there are no plans for consideration.

Junior doctors must be trained to identify the ultrasonographic features of PAS through continuous medical education, training, and mentoring.

- 4.Consultant Hematologist and Transfusion medicine specialist—Reserve 6 units of RCC, 6 units of platelets, 6 units of fresh frozen plasma, 30 units of cryoprecipitate. Rotation thromboelastometry (ROTEM) sample collection tube should be available in the theatre prior to surgery.The haematological laboratory must be informed regarding the probability of performing the ROTEM. The sample should be sent at times of excessive bleeding after quick liaison with the haematologist. Intravenous Tranexamic acid 1 g should be administered before the surgery.

Non-steroidal anti-inflammatory drugs should be avoided, and the patient should be provided with thromboembolic deterrent stockings.

Thromboprophylaxis should be initiated once the risk of bleeding is minimal. The selection must be case based after a MDT discussion.

- 5. Urologist- Cystoscopy facilities might be helpful in evaluating suspected bladder invasion. This facility should be established in future. Further, ureteric stenting facilities might be helpful in extreme cases.
- 6.Physiotherapist-. Commencing physiotherapy services at least 24 h prior to surgery improves the outcomes and thus chest and limb physiotherapy should be offered prior to surgery. Physiotherapy should be continued during hospital stay. Women should be advised on continuing physiotherapy until full recovery.
- 7. Nutritionist- Diet affects the post operative recovery of the patient. Prior referral enables patients to undergo screening for nutritional deficiencies,pre-operative loading of proteins and probiotics to promotewound healing.A high protein diet is started and continued once patient is surgically fit for oral intake.
- 8. Nursing officer- Patients will benefit from a dedicated PAS trained nursing officer. The nursing officer can educate the patient and discuss about the surgery, possible complications and what to anticipate during the convalescence period postoperatively. Tour of the ICU and neonatal care unit may be helpful to mitigate the stress response.

All stakeholders agreed to the check list for pre-operative and post-operative management. Furthermore, the psychiatrist must be a part of the team to provide counselling services before and after surgery and to assess mental wellbeing. Patients should be followed up for physical and psychological morbidity and their health status must be inquired biannually over the phone by a dedicated nurse.

## Discussion

PAS is a life-threatening situation associated with multiple physical and psychological complications. Mortality of 7% has been recorded in some case series [3]. However, in our study there were no maternal mortalities. All women with PAS were diagnosed prior to caesarean delivery.

During the last decade, many methods describing novel diagnostic modalities, prediction of surgical outcomes, novel techniques to reduce the blood loss and methods to preserve the uterus have been introduced [13–15]. However, not much attention has been paid to examine and improve the quality of life and psychological needs of women who are diagnosed with PAS.Interestingly, our study highlights inadequate justifications given for performing the primary CS in most of the women in our study. Indications such as ‘presumed macrosomia’, ‘non-engaged head at term’ and ‘maternal request’ need careful attention and re-evaluation. Increasing CS rates has become a worldwide issue as well as in Sri Lanka [10–12]. It is evident that a functional national governing body to audit CS is a necessity. Initiatives to use the Robson Classification is a considerable option [21, 22].

Vaginal birth after CS(VBAC) is at a lower rate in our study. Implementation of such practice is pivotal to mitigate complications such as PAS and RECS. We suggest mobilizing the support of social media, creating educational leaflets, and setting up of TOLAC dedicated nurses in the antenatal clinic, would promote and encourage women to make an informed decisions on opting for TOLAC. We also suggest that the testimonials and experiences of mothers who have had VBAC should be made available to other women before making their decision. Further, qualitative studies can be conducted with mother with successful VBAC and publish for the reference.

Health care stakeholders need to be motivated to provide facilities to establish this service by increasing facilities for monitoring (maternal and fetal) and arrange operating theatres close to the labour rooms in case of emergency during TOLAC. Root cause analysis for not offering TOLAC should be studied with its’ economic impact on the health care system. The cost for intervention and effectivity can be presented in budget meetings [23, 24].

The POA at diagnosis of PAS showed a wide range. Delay in diagnosis increases the risk to maternal and fetal life as life threatening bleeding can occur in early POA [25]. Early diagnosis reduces the risk and facilitates the early transfer to a tertiary center. Further, our participants claim that an early diagnosis allows the women to prepare for the surgery and its aftermath. 1^st^ trimester diagnosis of a possible PAS should be encouraged amongst the obstetricians [26–28].

MRI was not offered to any of the patients. However, its increased sensitivity aids in diagnosis when USS is inconclusive [29, 30]. Studies have shown that ultrasound scan is not a sensitive tool to diagnose posterior PAS and MRI should be considered in woman with posterior placenta previa presenting with other risk factors for PAS, such as curettage, even when the classical USS findings are not present [31]. Recent study has shown a weak correlation between FIGO and PAS score(ultrasound scoring systems) with histology specimen, Hence MRI has a role in planning for conservative surgery [32]. It is evident that in our setting the decision to perform hysterectomy or consevative suregry was taken intraoperatively. The availability of MRI might help in pre-operative assessment of the depth of invasion into the myometrium as well invasion of other organs and may increase the likelihood of conservative surgery.

Further, analysis of near miss cases has shown that PAS invasion in the lower uterine segment, bladder, and parametria were commonly missed [33]. These unanticipated PAS could lead to devastating consequences in LMIC although reported outcomes are satisfactory in other settings. Hence, good knowledge of various presentations of PAS, high level of suspicion, selective use of MRI to study the topography and depth of invasion and preparedness to handle unanticipated PAS can prevent undesirable outcomes in LMIC. Although MRI is widely used to assess the topography, there needs to be a standardized MRI staging system for PAS disorders [34].

The functionality of the PAS care bundle is evident at DMH. A multidisciplinary input, including the haematologist, transfusion medicine specialist, physiotherapist, and clinical nutritionist, might have contributed to the zero mortality and the absence of complications of massive blood transfusion. Psychiatrist and a dedicatd PAS care nurse should be included in the multidisciplinary team, and authors believe it might be beneficial in providing holistic care.

Blood loss during the surgery is reported to be higher than the other studies [35, 36]. New techniques such as early uterine artery clamping, usage of non-traumatic clamps and ligation of internal iliac arteries prior to commencement of hysterectomy in selected cases should be considered. The possibility of the novel triple P procedure which involves pre-operative positioning of arterial occlusion balloons and perioperative inflation to minimise blood loss during surgery should be explored though this might be challenging in a resource poor setting.

Uterine conservation has been a popular topic in the current decade. Furthermore, prediction models have also been developed to assess the outcomes. The external validity of these models are yet to be studied in LIMC as the outcome is determined by the performance of the team. Nevertheless, these studies are useful in counselling and decision making in women with PAS [16, 17].

A diagnosis of PAS and subsequent treatment is a stressful ordeal. The stress response is expected to reduce with time, yet some women were suffering from PTSD years after the surgery. Emotional instability of the mother can have dire repercussions within a family. Reduced scores in physical health and pain domains as elicited by the IES-R scoring, following surgery, affirms that the impact of PAS on the quality of life is dependent on subsequent physical and psychological wellbeing.. Long term follow-up and counselling of women suffering from poor physical health and pain following surgery can help in alleviating the symptoms. Patients were having chronic pelvic pain, back pain and dyspareunia which might be treatable with correct diagnostics. The SF-36 scoring also revealed that many domains are affected in these women irrespective of the time lapsed from the surgery.

Moreover, regular feedback from patients and team members might be helpful for quality improvement. Video recordings of the surgery need to be obtained and reviewed regularly to improve surgical techniques and skill.

A cost analysis was not carried out during the study and the authors believe that analysis of the relative costs of the procedures and care, along with expenditure for TOLAC would facilitate its promotion by relevant authorities. Partners of the women were not interviewed in the study. Their perspectives on the various issues faced by the women would further empower future research.

Our study was limited by the small number of patients, 27. A larger study may increase the yield of the issues identified or identify newer areas of concern. All women who were evaluated were diagnosed with PAS prior to surgery. The rate of complications may be higher in undiagnosed patients.

## Conclusions

Although the multidisciplinary team management of patients with PAS appears to be satisfactory, our study revealed that there is a major need for quality improvement. Further, hospital stay, and intraoperative blood loss seem to be higher in this setting. Patients are reported to have numerous physical and psychological difficulties in the long-term This requires continuous follow up of these patients providing medical and psychological support to cope with their problems.. However, some physical difficulties and psychological trauma may be lifelong. Implementing the ‘tailer made care package’ in our setting would be helpful in achieving desirable outcomes. Initiatives to minimizing primary CS rates and increasing TOLAC is a viable solution for saving the mothers’ lives and alleviating a lifelong burden. The implementation of the “tailor-made” care package in our setting would be helpful in achieving desirable outcomes. Further initiatives to minimise the primary CS rates and promoting VBAC are viable solutions for saving the lives of mothers with PAS and alleviating their lifelong burden.

## Supplementary Information

**Additional file 1.** Checklist for Placenta accreta spectrum.

## Data Availability

Original data cannot be shared with public due to restriction in ethical approval and research protocol. The corresponding author may share data upon request, after anonymizing the identity of patients within 5 years of the end of the study period.

## Abbreviations

PAS: Placenta Accreta Spectrum Disorder
CS: Caesarean section
LIMC: Low- andmiddle-income countries
APH: Antepartum Hemorrhage
MDT: Multidisciplinary team
GA: General Anesthesia
PTSD: Post-Traumatic stress disorder
DSHW: De Soysa Hospital for Women
IES-R: Impact of events scale (Revised)
INT: Intrusive subscale
AVD: Avoidance subscale
HYP: Arousal subscale
SF-S6: Short- form heath survey-36
ECS: Elective caesarean section
EmCS: Emergency caesarean section
RECS: Repeat elective caesarean section
TOLAC: Trial of labour after caesarean section
VBAC: Vaginal birth after caesarean section
POA: Period of amenorrhea
ICU: Intensive care units
DIC: Disseminated intravascular coagulation
SSI: Surgical site infection
EBL: Estimated blood loss
RCC: Red cell concentrate
USS: Ultrasound
ROTEM: Rotation thromboelastometry
