# Supplementary material for: Quality of surgical management of placenta accreta spectrum in a tertiary center in Sri Lanka: baseline study for quality improvement project: problems and solutions
Source: BMC Pregnancy Childbirth. 2022 Jun 23;22:509. doi: 10.1186/s12884-022-04840-7 (PMC9230081; doi:10.1186/s12884-022-04840-7)
Supplement: Supplementary file 1 — Additional file 1. Checklist for Placenta accreta spectrum. [file 12884_2022_4840_MOESM1_ESM.docx]

**Checklist**

| **Check list for Placenta Accreta spectrum** | | | | | | | |
| --- | --- | --- | --- | --- | --- | --- | --- |
| **Demographic data** | | | | | | | |
| Name |  | | | | | | |
| DOB |  | | Address | |  | | |
| Age |  | | Tel Number | |  | | |
| BHT |  | | Partners contact No | |  | | |
| Parity |  | | MOH area | |  | | |
| BMI |  | | Midwives’ Contact No | |  | | |
| Weight |  | | GP/MOH contact No | |  | | |
| Blood group |  | | Address | |  | | |
| Antibodies |  | | Tel Number | |  | | |
|  | | | | | | | |
| **Risk factors** | | | | | | | |
| Medical disorders | 1. …………………………………. 2. …………………………………. 3. …………………………………. 4. …………………………………. | | | | | | |
| Hematological disorders |  | | | | | | |
| Psychological disorders |  | | | | | | |
|  | | | | | | | |
| **Previous pregnancy details** | | | | | | | |
| pregnancy | MOD | | BW | | Age | | complications |
| P1 |  | |  | |  | |  |
| P2 |  | |  | |  | |  |
| P3 |  | |  | |  | |  |
| P4 |  | |  | |  | |  |
|  | | | | | | | |
| **Pre-op investigations** | | | | | **Results** | | **Date** |
| Hemoglobin | | | | |  | |  |
| Platelets | | | | |  | |  |
| PT-INR | | | | |  | |  |
| APTT | | | | |  | |  |
|  | | | | | | | |
| Thrombo- prophylaxis during the pre-op stay | | | | | YES | | NO |
| IF yes, DOSE | | | | |  | | |
|  | | | | | | | |
| **Diagnosis of PAS** | | | | | | | |
| POA of diagnosis | | |  | | | | |
| Diagnosed by | | |  | | | | |
| ***Criteria for diagnosis*** | | | | | | | |
| 1.Loss of the ‘clear zone’ | | | | | YES | | NO |
| 2.Abnormal placental lacunae | | | | | YES | | NO |
| 3.Bladder wall interruption | | | | | YES | | NO |
| 4.Myometrial thinning | | | | | YES | | NO |
| 5.Placental bulge | | | | | YES | | NO |
| 6.Focal exophytic masses | | | | | YES | | NO |
| 7.Uterovesical hypervascularity | | | | | YES | | NO |
| 8.Subplacental hypervascularity | | | | | YES | | NO |
| 9.Bridging vessels | | | | | YES | | NO |
| 10.Placental lacunae feeder vessels | | | | | YES | | NO |
|  | | | | | | | |
| Do you need MRI to diagnose | | | | | YES | | NO |
| Do suspect bladder involvement | | | | | YES | | NO |
| Do you suspect bowel involvement | | | | | YES | | NO |
|  | | | | | | | |
| **Setting of management** | | | | | | | |
| Need to Transfer to Tertiary care | | | | | YES | | NO |
|  | | | | | | | |
| **Antenatal complications** | | | | | YES | | NO |
| APH | | | | | YES | | NO |
| Blood transfusion | | | | | YES | | NO |
| Hospital stays more than 3 days | | | | | YES | | NO |
|  | | | | | | | |
| **Pre-operative measures** | | | | | | | |
| 1.Consultant obstetrician | | | Name | | Date | | Contact Number |
| Date of surgery | | |  | | | | |
| Type of surgery | | |  | | | | |
| Type of incision | | |  | | | | |
| Need for special instrument | | |  | | | | |
| Decision for sterilization in conservative | | |  | | | | |
| Consent form checked | | |  | | | | |
| Plan | | | | | | | |
| 2.Consultant Anesthetist | | | Name | | Date | | Contact Number |
| Type of anesthesia | | |  | | | | |
| Intravenous and arterial access | | |  | | | | |
| Post-operative pain relief | | |  | | | | |
| Post-op setting of management | | |  | | | | |
| Plan | | | | | | | |
| 3.Consultant Hematologist/transfusion medicine specialist | | | Name | | Date | | Contact Number |
| Blood and blood products | | |  | | | | |
| Timing of ROTEM | | |  | | | | |
| Plan | | | | | | | |
| 4.General/Colorectal surgeon | | | Name | | Date | | Contact Number |
| Need of bowel preparation | | |  | | | | |
| Plan | | | | | | | |
| 5.Consultant Urologist | | | Name | | Date | | Contact Number |
| Need of Stenting | | |  | | | | |
| Plan | | | | | | | |
| 6.Consultant Psychiatrist | | | Name | | Date | | Contact Number |
| Plan | | | | | | | |
| 7.Nutritioist | | | Name | | Date | | Contact Number |
| Plan | | | | | | | |
| 8.Physiotherapist | | | Name | | Date | | Contact Number |
| Plan | | | | | | | |
| 9.ICU care nurse | | | Name | | Date | | Contact Number |
| ICU tour/theatre tour | | |  | |  | |  |
| 10. Neonatologist | | | Name | | Date | | Contact Number |
| Dexamethasone | | |  | | | | |
| Plan | | | | | | | |
| 11. Consultant Radiologist | | | Name | | Date | | Contact Number |
| Do placental mapping before the surgery  Indicate the distance to placenta from bladder margin  Mark the fetal presentation (Cephalic/Breech/Oblique/Transverse) | | | | | | | |
|  | | | | | | | |
| Consent | | | Reviewed | | Date | | Sign |
| ICU booking | | | Checked | | Date | | Sign |
|  | | | | | | | |
| **Post-op summery** | | | | | | |  |
| **Surgery** | | | | | EMERGENCY | | ELECTIVE |
| POA | | | | | Week | | Dates |
| Surgery | | | | | Hysterectomy | | Conservative surgery |
| EBL | | | | | | | cc |
|  | | | | | | | |
| **Operative complications** | | | | | | | |
| 1.Bladder injury | | | | |  | |  |
| 2.Bowel injury | | | | |  | |  |
| 3.blood transfusions | | | | RCC | PLT | Cry | FFP |
| 4.ICU care | | | | |  | |  |
| 5.other (specify) | | | | |  | |  |
| 6.Other (Specify) | | | | |  | |  |
|  | | | | | | | |
| **Post- operative complications** | | | | | | | |
|  | | |  | |  | |  |
|  | | | | | | | |
| **Clearance for discharge with follow up plan** | | | | | | | |
| Obstetrician | | | Name | | Date | | Contact Number |
| Anesthetist | | | Name | | Date | | Contact Number |
| General surgeon/Urologist | | | Name | | Date | | Contact Number |
| Psychiatrist | | | Name | | Date | | Contact Number |
| Nutritionist | | | Name | | Date | | Contact Number |
| Physiotherapist | | | Name | | Date | | Contact Number |
|  | | | | | | | |
| **Follow ups** | | Date | Time | | Person in charge to arrange calls | | Any complications identified |
| 1 month | |  |  | |  | |  |
| 3months | |  |  | |  | |  |
| 6months | |  |  | |  | |  |
| 12 months | |  |  | |  | |  |
